# Immune-based disorders: the challenges for translational immunology

**DOI:** 10.1111/j.1582-4934.2008.00349.x

**Published:** 2008-04-15

**Authors:** David Pozo


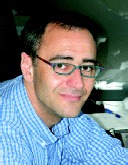


‘Translational therapies of immune-based disorders’ Review Series

Dear Editor,

Our immune system constantly interacts with our internal environment, protects us from our external environment and provides the inherent knowledge to sense the difference between friend and foe. Therefore, the immune system is one of the most dynamic body components in determining our state of health or disease. The importance of immune-based disorders is highlighted by the fact that immune-mediated inflammatory diseases are known to affect nearly 20% of the U.S. population. These conditions, which include juvenile-onset diabetes, rheumatoid arthritis, multiple sclerosis, asthma, systemic lupus erythematosus and acquired immune deficiency syndrome, lead to chronic debilitation, life-long illness, protracted morbidity and result in great medical costs. The immune system also has a major involvement in post-transplant rejection and in the body's ability to fight invading cancer cells.

Despite of these seemingly unconnected diseases, basic immunologists are in a prominent position to apprehend a broader picture under a cohesive paradigm. This forefront of future therapeutic strategies relies on the successful applications of genetic, molecular and cellular immunology to prevent, treat and cure the problems caused by immune-based disorders. The translational aspects of research that are directly related to patient care require integrated, multidisciplinary strategies involving teams skilled in basic biology and clinical investigation, ethics and regulatory issues. To take full advantage of such a global approach, it is essential to focus research programs on a limited number of well-validated basic concepts common to autoimmunity, allergy and organ transplantation. Thus, how to best enforce or end immune responses for the benefit of the patients is probably the most exciting attainable goal for today's therapeutics [1].

Starting with this issue, we are pleased to launch a new review series in the *Journal of Cellular and Molecular Medicine*, devoted to present and discuss new approaches of potential relevance for the development of translational immunology. Better treatments, prevention and a cure, are the ultimate goals of multiple sclerosis (MS) research, and perhaps no branch of investigation has proven more fruitful towards these goals than the study of the immune system. In the first review article appearing in this issue, Quintana, Farez and Weiner at the Harvard University Center for Neurologic Diseases, explore MS when complex data from multiple experimental sources using interdisciplinary tools are integrated and analyzed [2–4]. Current therapies for MS have emerged from our growing understanding of how the immune system works and how it can be manipulated to suppress or regulate immune attacks. Here, they discuss how the development of computational tools for the integration of multiple data into quantitative models [5, 6], represents one of the last frontiers in drug discovery programs and the early detection of individuals at risk of suffering MS. The series will go on with key questions with a clear emphasis on translational issues. Some of them are as follows: What is the future of novel biochemical pathways and lipid-derived mediators as anti-inflammatory modulatory agents? Which are the limits posed by the immune system to different gene therapy strategies? What are the immune aspects of amyloidosis-based diseases? What are the immunological problems and the prospective solutions for stem cell-based therapies? What can be learned about the immune regulation of human haematopoietic stem cells? What are the translational values of the immunomodulatory capacities of mesenchymal stem cells? These, among other issues, will highlight ongoing research to bring forth valuable points and new ideas for discussion. Your comments, suggestions and contributions aimed to integrate basic and clinical immunology, will be warmly welcomed.
